# Posterior hippocampal CA2/3 volume is associated with autobiographical memory recall ability in lower performing individuals

**DOI:** 10.1038/s41598-023-35127-2

**Published:** 2023-05-16

**Authors:** Ian A. Clark, Marshall A. Dalton, Eleanor A. Maguire

**Affiliations:** 1 Wellcome Centre for Human Neuroimaging, Department of Imaging Neuroscience, UCL Queen Square Institute of Neurology, University College London, London, UK; 2 School of Psychology, The University of Sydney, Sydney, Australia

**Keywords:** Cognitive neuroscience, Learning and memory

## Abstract

People vary substantially in their capacity to recall past experiences, known as autobiographical memories. Here we investigated whether the volumes of specific hippocampal subfields were associated with autobiographical memory retrieval ability. We manually segmented the full length of the two hippocampi in 201 healthy young adults into DG/CA4, CA2/3, CA1, subiculum, pre/parasubiculum and uncus, in the largest such manually segmented subfield sample yet reported. Across the group we found no evidence for an association between any subfield volume and autobiographical memory recall ability. However, when participants were assigned to lower and higher performing groups based on their memory recall scores, we found that bilateral CA2/3 volume was significantly and positively associated with autobiographical memory recall performance specifically in the lower performing group. We further observed that this effect was attributable to posterior CA2/3. By contrast, semantic details from autobiographical memories, and performance on a range of laboratory-based memory tests, did not correlate with CA2/3 volume. Overall, our findings highlight that posterior CA2/3 may be particularly pertinent for autobiographical memory recall. They also reveal that there may not be direct one-to-one mapping of posterior CA2/3 volume with autobiographical memory ability, with size mattering perhaps only in those with poorer memory recall.

## Introduction

The ability to recall autobiographical memories of our past experiences varies substantially across healthy individuals. While some people can recollect decades-old autobiographical memories in great detail, others struggle to recall what they did last weekend^1–3^. The hippocampus is central to the processing of autobiographical memories; when it is damaged and its volume reduced, this typically results in autobiographical memory recall impairments^4–7^. However, no consistent relationship between autobiographical memory recall ability and hippocampal grey matter volume in healthy young adults is evident^1,8–10^.

This may be because measurements of whole hippocampal volume lack the nuance required to detect structure–function relationships in healthy people, given that the hippocampus is not a homogenous structure. It comprises anatomically distinct subregions—the dentate gyrus (DG), Cornu Ammonis (CA) 1–4, the subiculum, the presubiculum, the parasubiculum and an anatomically complex region called the uncus^11–13^—each with different connections to other brain areas^14–20^.

Investigations into human hippocampal subfields and autobiographical memory are relatively few and have been limited to small sample sizes because it is challenging to delineate subfields from structural magnetic resonance image (MRI) scans. While automated methods are available^21^, the considerable inter-subject variability in the morphology of the hippocampus makes it difficult to achieve sufficient accuracy, especially along the full length of the hippocampus. Consequently, manual delineation of hippocampal subfields remains the gold standard^22^, which requires expertise and is time-consuming, especially if performed at scale.

Considering existing evidence concerning human hippocampal subfields and autobiographical memory, damage that appears to be specific to individual subfields, such as CA2/3^23^ or CA1^24^, can result in compromised autobiographical memory recall. In functional MRI (fMRI) studies, the DG and CA2/3 subfields, particularly in their posterior portions, were found to support representations of remote (10 years old) autobiographical memories^25^. More anteriorly, the presubiculum and parasubiculum (typically combined into one region of interest (ROI) called the pre/parasubiculum) are often activated during autobiographical memory recall^26,27^.

Preliminary evidence also suggests that variation in the volume of specific subfields may be related to autobiographical memory recall ability. In one study, left pre/parasubiculum volume was found to correlate with autobiographical memory persistence over an eight month delay^28^. Another study focused solely on the body, or mid portion, of the hippocampus, and reported that the volume of a combined ROI comprising the left DG/CA4/CA2/3, as well as a bilateral ROI where subiculum and pre/parasubiculum were combined, were correlated with autobiographical memory recall ability^29^.

This latter study has particular relevance for our current experiment, but several of its features should be noted. First, as alluded to above, only a coarse segmentation of subfields was performed. The hippocampus was divided into three sections: a combined DG/CA4/CA2/3 region, CA1, and a combined subiculum and pre/parasubiculum region. This could be problematic given the identification of differential memory findings for DG compared to CA2/3^23,30–32^, and the specific association of the pre/parasubiculum, as opposed to the subiculum, with autobiographical memory recall^26,28,33^. Moreover, subfield segmentation was performed only for the body of the hippocampus. While the hippocampal body is easier to segment than the anterior and most posterior portions of the hippocampus, it makes up less than half the total hippocampal volume^34^. Consequently, focusing solely on the body ignores differences in the subfields along the entire long axis of the hippocampus^11,19,25,33,35^. Second, only 30 participants were studied. Small sample sizes are a common limitation when studying hippocampal subfields, and it has been suggested that investigations into structure–function relationships in healthy people require sample sizes in the hundreds in order to provide reliable results^36,37^. Third, while autobiographical memory recall was examined, there was no control task. This makes it difficult to know whether significant subfield associations were specific to autobiographical memory recall.

Overall, the very small number of studies examining subfield involvement in autobiographical memory recall suggest that DG and/or CA3, and subiculum and/or pre/parasubiculum may have particular relevance. However, there is clearly a need for larger-scale, more spatially-resolved studies. Consequently, here we aimed to comprehensively investigate the relationship between hippocampal subfield volumes and autobiographical memory recall ability in healthy people.

**Figure 1:**
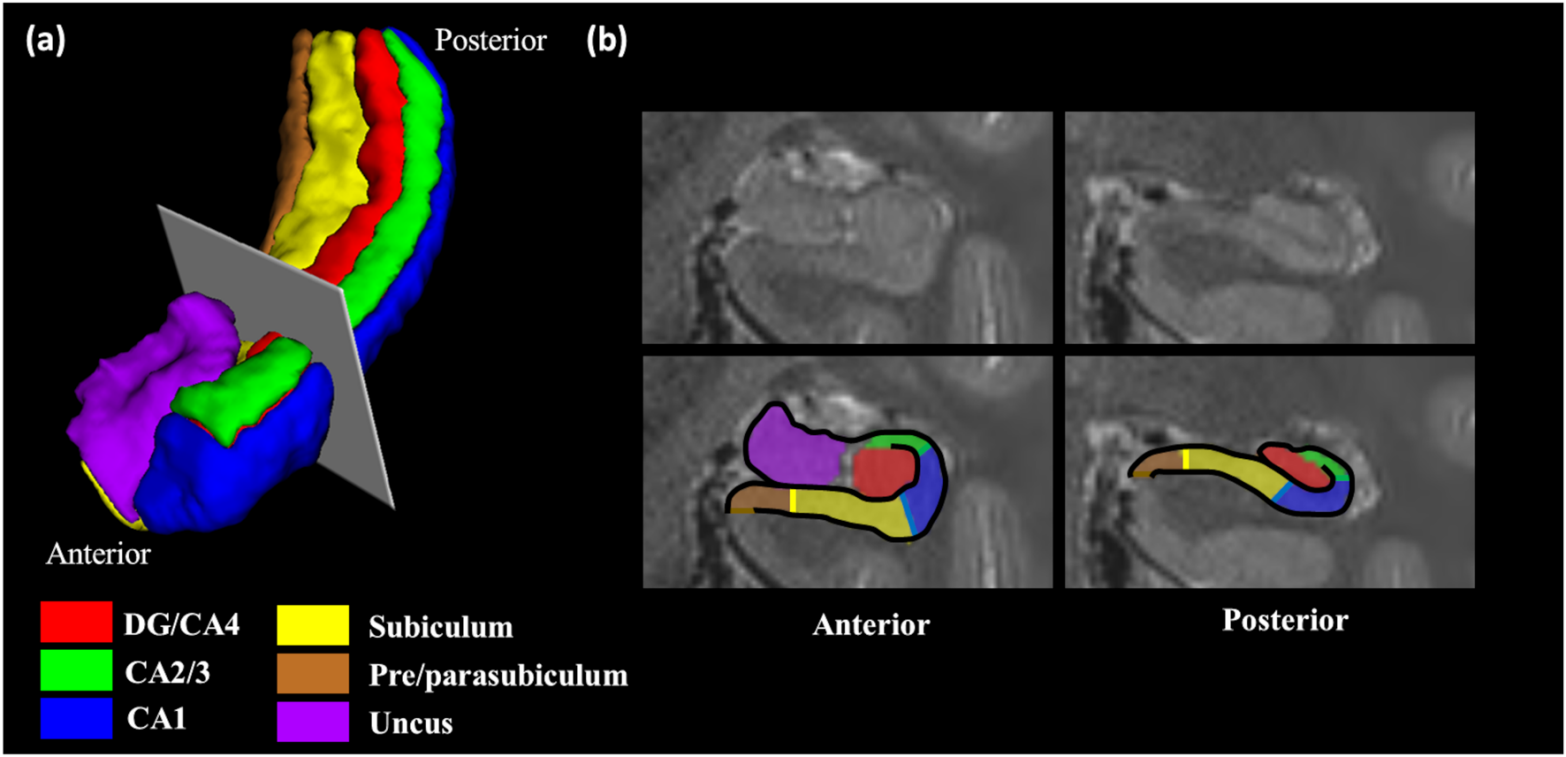
The hippocampal subregions examined in this study. (**a**) A 3D representation, viewed from an antero-lateral perspective, of the segmented hippocampal subfields, and the boundary used to divide the anterior and posterior portions. (**b**) Two sections from T2-weighted structural MRI scans of an example hippocampus (top panel) overlaid with hippocampal subfield masks (lower panel). The left image is from the anterior hippocampus, and the right image from the posterior hippocampus.

Hippocampal subfield segmentation was performed using the detailed methodology described by Dalton et al.^22^, which involved manually delineating subfields along the full length of the hippocampus into six subregions: DG/CA4, CA2/3, CA1, subiculum, pre/parasubiculum and uncus (Fig. 1). This protocol permits separation of the DG/CA4 from CA2/3, as well as dividing the subicular cortices into the subiculum and pre/parasubiculum. As the full length of the hippocampus was segmented, each subfield could also be divided into their anterior and posterior portions^38,39^, with the exception of the uncus which lies solely in the anterior hippocampus.

To ensure an appropriate sample size and a wide range of autobiographical memory recall ability, 201 healthy young adults participated who were recruited from the general population (104 female, 97 male; mean age of 29.05 years, *SD* = 5.65; age was restricted to between 20 and 41 years to limit the possible effects of aging). Their 402 hippocampi were manually segmented (each person’s hippocampi took ~ 8 h to segment). Participants also underwent the widely-used Autobiographical Interview^40^, which provided a metric for their autobiographical memory recall ability (episodic internal details) as well as a control measure (semantic details). Ideally, we would have also included an additional widely-used independent measure of autobiographical memory recall ability. However, other than the Autobiographical Interview, there is a dearth of such measures. In addition, we examined performance on eight standard laboratory-based memory tests. This allowed us to investigate whether any relationships with subfield volumes were specific, or not, to recollecting autobiographical memories from real life. This is pertinent because it has been shown that the neural correlates of recollecting autobiographical memories of real lived experiences differ substantially from those arising from laboratory-based memory recall tasks^41^. A similar distinction was recently reported where the influence of specific white matter microstructure features on axonal conduction velocity was evident when recalling autobiographical memories, but not laboratory-based memory stimuli^42^.

The analyses reported here mirror those in our previous published studies investigating relationships between the hippocampus and autobiographical memory ability^8,43^. This approach is designed to reflect the range of methods pursued in the literature, allowing us to perform a broad and inclusive evaluation of any associations between the hippocampus and autobiographical memory recall performance. Specifically, we first typically examine the relationship between hippocampal volume and autobiographical memory recall ability using the full sample. Next, we investigate male and female participants to examine possible effects of gender. Following this, we perform a median split and partial correlations in each of the lower and higher performing groups. We then compare directly the volumes of lower and higher performing groups. Finally, we focus on only the very highest and lowest performers (usually n = 20 in each group), comparing volumes of these groups who represent the extremes of performance. In the current study, we adhere to the same approach, but instead of considering whole hippocampal volume, we now examine the volume of each hippocampal subfield (whole, anterior and posterior) and its relationship, if any, with autobiographical memory recall ability.

Considering the limited extant literature outlined above, we predicted that CA2/3 and/or DG/CA4, and subiculum and/or pre/parasubiculum volumes would be the most likely to associate with autobiographical memory recall ability^28,29^. We also hypothesised that relationships with DG/CA4 and/or CA2/3 would localise to the posterior hippocampus^25^, while relationships with the pre/parasubiculum would localise to the anterior hippocampus^26,27^. Finally, we predicted that any relationships identified with subfield volumes would be specific to autobiographical memory recall rather than laboratory-based memory measures^41,42^.

## Results

As in previous studies^8,43^, planned analyses first involved performing partial correlations to investigate relationships between hippocampal subfield volumes and autobiographical memory recall in the whole group (n = 201), then in male (n = 97) and female (n = 104) participants separately, and then in the lower (n = 101) and higher (n = 100) performing groups (identified by performing a median split based on the main autobiographical memory recall measure). Univariate ANCOVAs were then used to compare the hippocampal subfield volumes of the lower and higher performing groups, with a similar approach also used for the highest and lowest performing participants (n = 20 in each). Six covariates were included in each analysis: age, gender, full scale IQ, scanner, total hippocampal volume (or total anterior or posterior hippocampal volume when examining anterior or posterior subfield volumes) and total intracranial volume. The exceptions were the analyses investigating male and female participants separately, where gender was not included as a covariate. The results for left and right hippocampi were very similar, so here we report bilateral hippocampal findings. As we investigated six hippocampal subfields, and two main performance measures of interest (internal details and semantic details from the Autobiographical Interview^40^), we corrected for this using the Bonferroni method—dividing alpha = 0.05 by 12. Consequently, only associations with two-sided *p* < 0.0042 were considered statistically significant.

### Autobiographical memory recall scores

Autobiographical memory recall ability was examined with the widely-used Autobiographical Interview^40^ (see “Methods”). The main measure was the mean number of “internal” details; these describe the specific past event in question, and are considered to reflect episodic information. Across participants, the mean number of internal details provided per memory was 24.03 (*SD* = 7.27; range = 4.60–44.60). In male participants, the mean number of internal details per memory was 23.12 (*SD* = 7.38; range = 10.2–44) and for females it was 24.88 (*SD* = 7.08; range = 4.60–44.60). After participants were allocated to the lower or higher performing groups based on a median split (see Methods), the mean number of internal details per memory for the lower group was 18.22 (*SD* = 3.59; range = 4.60–23.4), and for the higher group was 29.89 (*SD* = 4.94; range = 23.60–44.60). In the highest performing participants (see Methods), the mean number of internal details per memory was 37.83 (*SD* = 2.98; range = 34.60–44.60), and in the lowest performers it was 13.0 (*SD* = 2.58; range = 4.60–15.20).

In line with Mair et al.^44^, see also^29,45^, a control measure from this task, the mean number of semantic details per memory, was also calculated from the autobiographical memory descriptions. Semantic details pertaining to a past event are not considered to reflect autobiographical memory recall ability. Across the whole group, the mean number of semantic details provided per memory was 2.71 (*SD* = 1.57; range = 0–8.0), for male participants it was 2.72 (*SD* = 1.33; range = 0.40–6.20), for female participants it was 2.70 (*SD* = 1.75; range = 0.0–8.0), for lower performers it was 2.65 (*SD* = 1.51; range = 0.4–8.0), for higher performers it was 2.78 (*SD* = 1.61; range 0–7.0), and finally, for the highest performers it was 3.38 (*SD* = 1.32; range = 1.0–6.20) and for the lowest performers it was 1.86 (*SD* = 0.84; range = 0.40–3.60).

### Hippocampal subfield volumes

Mean bilateral volumes for the hippocampal subfields were as follows: DG/CA4 = 1142.73 mm^3^ (*SD* = 157.75 mm^3^, range = 785.73–1686.06 mm^3^); CA2/3 = 301.26 mm^3^ (*SD* = 50.89 mm^3^, range = 184.73–458.44 mm^3^); CA1 = 1239.40 mm^3^ (*SD* = 157.36 mm^3^, range = 903.70–1816.0 mm^3^); subiculum = 1110.16 mm^3^ (*SD* = 164.83 mm^3^, range = 665.40–1630.0 mm^3^); pre/parasubiculum = 541.58 mm^3^ (*SD* = 84.56 mm^3^, range = 364.0–812.2 mm^3^); uncus = 888.54 mm^3^ (*SD* = 192.09 mm^3^, range = 526.0–1579.0 mm^3^).

### No relationships between autobiographical memory recall ability and hippocampal subfield volumes across the whole group

We first investigated whether autobiographical memory recall ability was associated with the volume of each hippocampal subfield across all participants. No significant relationships were identified for any subfield (Table 1). In addition, no significant relationships were evident when examining the anterior and posterior portions of each subfield separately (Supplementary Tables S1 and S2).

**Table 1:** Partial correlations between autobiographical memory recall ability (internal details) and bilateral hippocampal subfield volumes across the whole group.

Hippocampal subfield	r(193)	p	95% Confidence interval	
			Lower	Upper
DG/CA4	− 0.08	0.29	− 0.22	0.06
CA2/3	0.10	0.15	− 0.04	0.24
CA1	− 0.10	0.18	− 0.23	0.04
Subiculum	0.10	0.18	− 0.04	0.23
Pre/parasubiculum	0.11	0.12	− 0.03	0.25
Uncus	− 0.03	0.66	− 0.17	0.11

Threshold for statistical significance is *p* < 0.0042 (Bonferroni correction for multiple comparisons).

### No relationships between autobiographical memory recall ability and hippocampal subfield volumes in male and female participants

We next examined whether autobiographical memory recall ability was associated with the volume of each hippocampal subfield in male or female participants separately. No significant relationships were identified for any subfield (Table 2).

**Table 2:** Mean volumes and partial correlations—autobiographical memory recall ability (internal details) and bilateral hippocampal subfield volumes in male and female participants.

Hippocampal subfield	Mean (SD) volume (mm^3^)	r	p	95% Confidence interval	
				Lower	Upper
Male participants		r(90)			
DG/CA4	1146.59 (163.86)	− 0.14	0.19	− 0.33	0.07
CA2/3	301.84 (56.24)	0.20	0.06	− 0.00	0.39
CA1	1267.84 (168.87)	− 0.07	0.52	− 0.27	0.14
Subiculum	1137.17 (169.62)	0.00	1.0	− 0.20	0.20
Pre/parasubiculum	565.78 (90.19)	0.13	0.21	− 0.08	0.33
Uncus	969.88 (191.95)	0.04	0.74	− 0.17	0.24
Female participants		r(97)			
DG/CA4	1139.13 (152.53)	− 0.01	0.96	− 0.20	0.19
CA2/3	300.72 (45.61)	− 0.02	0.88	− 0.21	0.18
CA1	1212.89 (141.53)	− 0.12	0.25	− 0.31	0.08
Subiculum	1084.96 (156.88)	0.20	0.04	0.01	0.39
Pre/parasubiculum	519.01 (72.37)	0.07	0.50	− 0.13	0.26
Uncus	812.68 (158.94)	− 0.12	0.23	− 0.31	0.08

Threshold for statistical significance is *p* < 0.0042 (Bonferroni correction for multiple comparisons).

### CA2/3 volume was associated with autobiographical memory recall ability specifically in lower performing individuals

**Figure 2:**
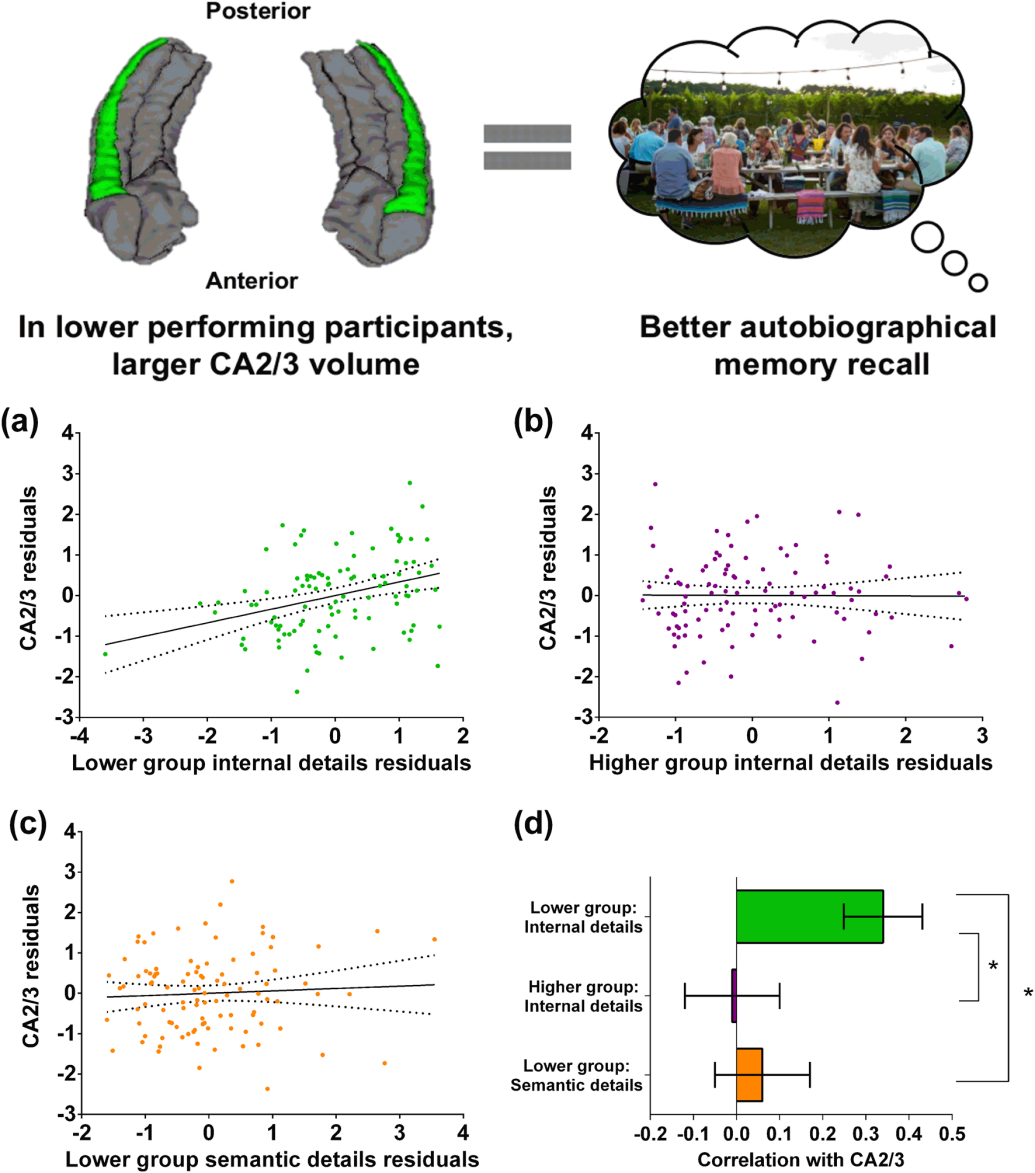
CA2/3 volume and autobiographical memory recall ability. (**a**) There was a significant positive correlation between bilateral CA2/3 volume and internal details in the lower performing group (dashed lines indicate the confidence intervals). (**b**) There was no significant relationship between bilateral CA2/3 volume and internal details in the higher performing group. (**c**) There was no significant relationship between bilateral CA2/3 volume and semantic details in the lower performing group. (**d**) The bar chart shows the partial correlation coefficients (with standard errors) between bilateral CA2/3 volume and internal details in the lower (green) and higher (magenta) performing groups, and with semantic details (orange) in the lower performing group. There was a significant difference between the correlations when they were directly compared; *p < 0.05. The free to use photograph is by David Todd McCarty on Unsplash (https://unsplash.com/license).

Partial correlations were performed separately for the lower and higher scoring groups, and a significant relationship with one subfield was found. There was a positive association between CA2/3 volume and autobiographical memory recall ability specifically in the lower performing group (Fig. 2a; *r*(93) = 0.34, *p* = 0.0009, 95% CI 0.14, 0.50). There was no such association evident for the higher performing group (Fig. 2b; *r*(92) = − 0.01, *p* = 0.95, 95% CI − 0.21, 0.20). Direct comparison of the correlations (two-sided, *p* < 0.05, see Methods) confirmed there was a significantly larger correlation in the lower performing group than the higher performing group (Fig. 2d; mean r difference = 0.35 (95% CI 0.07, 0.60), *z* = 2.52, *p* = 0.012). No other correlations that survived Bonferroni correction were observed in the other subfields (Table 3). For completeness, the correlations between CA2/3 volume and each of the internal detail sub-categories in the lower performing group are provided in Supplementary Table S3 with no statistically significant results. Similarly, Supplementary Table S4 shows the correlations, in the lower performing group, between CA2/3 volume and autobiographical memory recall ability for each of the four time periods included in the Autobiographical Interview (early childhood, teenage years, adulthood and from the last year), with no statistically significant results.

**Table 3:** Mean volumes and partial correlations—autobiographical memory recall ability (internal details) and bilateral hippocampal subfield volumes when split into lower and higher performing groups.

Hippocampal subfield	Mean (SD) volume (mm^3^)	r	p	95% Confidence interval	
				Lower	Upper
Lower performing group		r(93)			
DG/CA4	1154.41 (153.39)	0.26	0.012	0.06	0.43
CA2/3	299.53 (46.95)	0.34	0.0009*	0.14	0.50
CA1	1243.61 (156.19)	− 0.24	0.019	− 0.42	− 0.04
Subiculum	1105.93 (172.93)	− 0.19	0.07	− 0.37	0.02
Pre/parasubiculum	536.41 (86.16)	0.09	0.41	− 0.12	0.28
Uncus	899.32 (185.98)	0.00	0.97	− 0.20	0.21
Higher performing group		r(92)			
DG/CA4	1130.93 (161.94)	− 0.11	0.27	− 0.31	0.09
CA2/3	303.0 (54.76)	− 0.01	0.95	− 0.21	0.20
CA1	1235.16 (159.20)	− 0.06	0.60	− 0.26	0.15
Subiculum	1114.42 (156.99)	0.21	0.04	0.01	0.40
Pre/parasubiculum	546.81 (83.02)	− 0.02	0.83	− 0.22	0.18
Uncus	877.66 (198.41)	− 0.03	0.75	− 0.23	0.17

*Significant after Bonferroni correction (thresholded at *p* < 0.0042).

To test whether the observed relationship with CA2/3 volume in the lower performing participants was specific to internal (episodic) details of the autobiographical memories, we performed a further partial correlation analysis using the semantic details control measure. No association was evident between CA2/3 volume and the number of semantic details (Fig. 2c; *r*(93) = 0.06, p = 0.57, 95% CI − 0.14, 0.26). Direct comparison of the correlations confirmed that in the lower performing group there was a significantly larger correlation between CA2/3 volume and internal details than for semantic details (Fig. 2d; mean r difference = 0.28 (95% CI 0.06, 0.53), *z* = 2.45, *p* = 0.014).

### Effects were localised to posterior CA2/3

**Figure 3:**
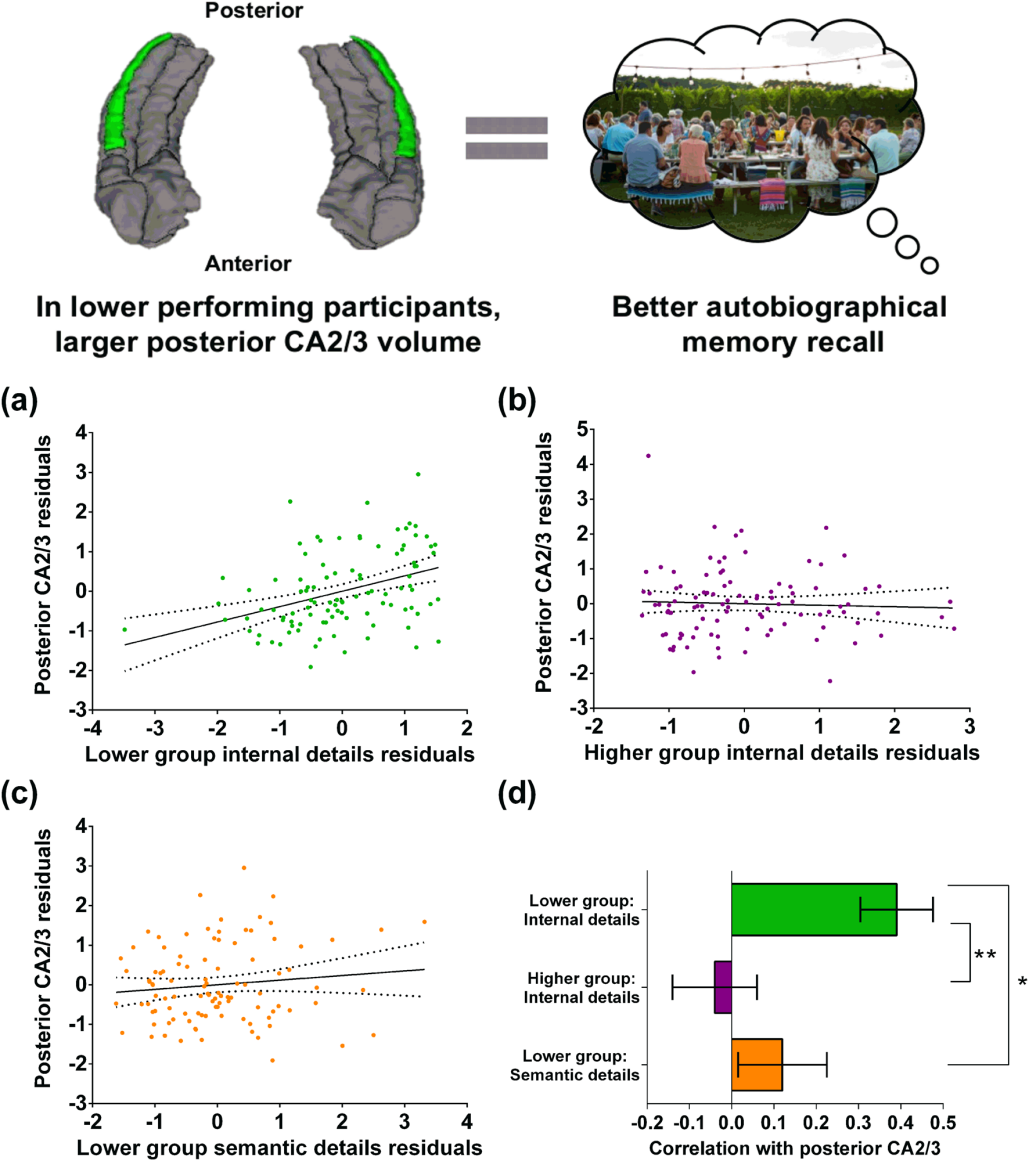
Posterior CA2/3 volume and autobiographical memory recall ability. (**a**) There was a significant positive correlation between bilateral posterior CA2/3 volume and internal details in the lower performing group (dashed lines indicate the confidence intervals). (**b**) There was no significant relationship between bilateral posterior CA2/3 volume and internal details in the higher performing group. (**c**) There was no significant relationship between bilateral posterior CA2/3 volume and semantic details in the lower performing group. (**d**) The bar chart shows the partial correlation coefficients (with standard errors) between bilateral posterior CA2/3 volume and internal details in the lower (green) and higher (magenta) performing groups, and with semantic details (orange) in the lower performing group. There was a significant difference between the correlations when they were directly compared; **p < 0.01, *p < 0.05. The free to use photograph is by David Todd McCarty on Unsplash (https://unsplash.com/license).

We next examined anterior and posterior volumes to ascertain whether or not a particular portion of CA2/3 was driving the effect we observed in the lower performing group. No significant relationship was identified between anterior CA2/3 volume and autobiographical memory recall (*r*(93) = 0.18, *p* = 0.08, 95% CI− 0.02, 0.37). By contrast, a significant positive relationship was evident between posterior CA2/3 volume and autobiographical memory recall (Fig. 3a; *r*(93) = 0.39, *p* = 0.0001, 95% CI 0.20, 0.55). No relationship between anterior CA2/3 volume (*r*(92) = 0.05, *p* = 0.62, 95% CI − 0.15, 0.25) or posterior CA2/3 volume (Fig. 3b; *r*(92) = − 0.04, *p* = 0.67, 95% CI − 0.24, 0.16) and autobiographical memory recall in the higher performing group was evident. Direct comparison of the posterior CA2/3 correlations confirmed there was a significantly larger correlation with autobiographical memory recall ability in the lower performing group than the higher performing group (Fig. 3d; mean r difference = 0.43 (95% CI 0.16, 0.68), *z* = 3.15, *p* = 0.0016). For completeness, the correlations between posterior CA2/3 volume and each of the internal detail sub-categories in the lower performing group are provided in Supplementary Table S5. We note that the “events” sub-category was close to being statistically significant. Supplementary Table S6 shows the correlations, in the lower performing group, between posterior CA2/3 volume and autobiographical memory recall ability for each of the four time periods included in the Autobiographical Interview, with no statistically significant results.

In addition, no relationship was identified between posterior CA2/3 volume and the semantic details control measure (Fig. 3c; *r*(93) = 0.12, *p* = 0.26, 95% CI − 0.09, 0.31) for the lower performing group. Direct comparison of the correlations confirmed a significantly larger correlation between posterior CA2/3 volume and internal details than with semantic details in the lower performing group (Fig. 3d; mean r difference = 0.27 (95% CI 0.05, 0.53), *z* = 2.40, *p* = 0.016).

### The relationship with posterior CA2/3 volume in lower performing participants was specific to autobiographical memory recall

As noted above, the posterior CA2/3 finding for the lower performing group was apparent only for internal (episodic) details and not for semantic details from the same autobiographical memories. This suggests that the effect might be specific to internal details. To investigate this further, we also examined whether there were any other measures that distinguished between the lower and higher performing groups and, if so, whether they also had a statistically significant relationship with posterior CA2/3 volume.

As shown in Table 4, the lower and higher performing groups were well matched. No statistically significant differences were apparent in age, gender, the distribution across the scanners used or full scale IQ^46^. Moreover, there were no statistically significant differences between the groups on a range of laboratory-based memory tasks, including tests of their verbal list recall ability, assessed using the immediate and delayed recall of the Rey Auditory Verbal Learning Test^47^, their visuospatial recall ability, examined using the delayed recall of the Rey–Osterrieth Complex Figure^48^, their recognition memory ability, tested using the Warrington Recognition Memory Tests for Words and Faces^49^, and on the “Dead or Alive” task, which probes general knowledge about whether famous people have died or are still alive, providing a measure of semantic memory^50^.

**Table 4:** Comparison of the lower and higher performing groups on other measures.

	Lower performing			Higher performing			Test	p
	S1	S2	S3	S1	S2	S3	χ^2^ (2)	
Number of participants on each scanner	38	27	36	27	40	33	4.51	0.11

	M		F	M		F	χ^2^ (1)	
Gender	55		46	42		58	3.12	0.077

	Mean		SD	SD	Mean		t(199)	
Age	28.86		6.04	5.26	29.24		0.47	0.64
FSIQ	101.83		7.70	7.63	103.88		1.89	0.06
RAVLT immediate (aggregate) recall	58.40		8.10	6.62	59.16		0.73	0.47
RAVLT delayed recall	12.77		2.27	1.99	13.12		1.16	0.25
Warrington RMT for words	12.44		2.36	1.81	12.93		1.63	0.10
Warrington RMT for faces	10.66		3.31	3.36	11.36		1.48	0.14
Rey figure delayed recall	21.99		5.28	6.11	22.76		0.96	0.34
Dead or alive proportion correct	80.44		8.08	8.22	82.39		1.70	0.09
Logical memory immediate recall	12.44		2.24	1.74	13.53		3.87	0.00015*
Logical memory delayed recall	11.77		2.71	2.18	13.42		4.74	< 0.0001*

*S* scanner, *M* male, *F* female, *FSIQ* Full Scale Intelligence Quotient estimated from the Test of Premorbid Functioning, *RAVLT* Rey Auditory Verbal Learning Test, *RMT* Recognition Memory Test, *Rey Figure* Rey–Osterrieth Complex Figure. For the Warrington RMTs and the Logical Memory tests the scaled scores are reported. *Indicates a significant difference between the two groups.

Differences were, however, observed between the lower and higher performing groups on the immediate and delayed recall Logical Memory subtest of the Wechsler Memory Scale IV^51^. This test assesses a participant’s ability to recall a short narrative and can be considered the closest of the laboratory-based memory retrieval tests we examined to autobiographical memory recall. However, performing partial correlation analyses in the lower performing participants showed that there were no significant relationships between posterior CA2/3 volume and immediate or delayed recall performance of the Logical Memory test (immediate recall: *r*(93) = − 0.01, p = 0.90, 95% CI − 0.21, 0.19; delayed recall: *r*(93) = − 0.13, p = 0.21, 95% CI − 0.32, 0.07). Direct comparison of the correlations confirmed that there was a significantly larger correlation between posterior CA2/3 volume and autobiographical memory internal details than for the immediate (mean r difference = 0.40, 95% CI 0.13, 0.71, z = 2.86, p = 0.0043) and delayed (mean r difference = 0.52, 95% CI 0.25, 0.83, z = 3.67, p = 0.0002) recall of the Logical Memory test in the lower performing group.

### Subfield volumes did not differ across performance groups

We next investigated whether subfield volumes differed between the lower and higher performing groups, or between the very highest and lowest performers (n = 20 in each group). No differences were identified for any subfield (Table 5).

**Table 5:** Comparisons of the mean bilateral hippocampal subfield volumes (mm^3^) between the lower and higher performing groups and the very highest and lowest performers.

Hippocampal subfield	Mean	SD	Mean	SD	Test	p
	Lower performing		Higher performing		F(1,192)	
DG/CA4	1154.41	153.39	1130.93	161.94	2.61	0.11
CA2/3	299.53	46.95	303.0	54.76	0.24	0.62
CA1	1243.61	156.19	1235.16	159.20	0.07	0.80
Subiculum	1105.93	172.93	1114.42	156.99	1.70	0.19
Pre/parasubiculum	536.41	86.16	546.81	83.02	2.15	0.14
Uncus	899.32	185.98	877.66	198.41	0.21	0.65

	Very lowest performers		Very highest performers		F(1,31)	
DG/CA4	1128.13	159.15	1123.85	124.36	0.41	0.53
CA2/3	288.71	47.13	306.17	58.73	3.60	0.07
CA1	1327.49	199.67	1237.27	153.07	3.14	0.09
Subiculum	1174.63	171.82	1176.97	152.49	0.80	0.38
Pre/parasubiculum	555.76	98.50	557.42	67.93	0.05	0.82
Uncus	969.90	168.86	934.64	228.16	0.04	0.84

Threshold for statistical significance is *p* < 0.0042 (Bonferroni correction for multiple comparisons).

As the significant relationship in the lower performing group was localised to posterior CA2/3, we also compared the posterior CA2/3 volumes between the lower and higher performing groups and found no difference between them (lower performing mean volume = 202.05 mm^3^, *SD* = 34.25; higher performing mean volume = 206.10 mm^3^, *SD* = 39.84; F(1,192) = 0.78, p = 0.38). This suggests that there may not be a simple linear relationship between posterior CA2/3 volume and autobiographical memory ability. To investigate this further, we performed an additional analysis to help inform the interpretation of the results. Instead of dividing the participants into two groups based on their autobiographical memory internal details scores, we instead used the posterior CA2/3 hippocampal volumes to median split participants into smaller volume and larger volume groups. When correlated with the autobiographical memory recall scores, no statistically significant relationship was found for either group (smaller volume group: *r*(93) = 0.25, *p* = 0.01, 95% CI 0.05, 0.43; larger volume group: *r*(92) = 0.16, *p* = 0.13, 95% CI − 0.05, 0.35).

## Discussion

In this individual differences study, we manually segmented the full length of the two hippocampi in 201 healthy individuals. We delineated six subregions—DG/CA4, CA2/3, CA1, subiculum, pre/parasubiculum and uncus. This allowed us to examine how hippocampal subfield volumes might relate to autobiographical memory recall ability in the most comprehensive manner attempted to date. Across the group we found no evidence for an association between the volume of any subfield and autobiographical memory recall ability as measured using internal details from the Autobiographical Interview^40^. This was also the case when investigating male and female participants separately. However, when participants were assigned to lower and higher performing groups based on their internal details scores, we found that CA2/3 volume was significantly and positively associated with autobiographical memory recall performance specifically in the lower performing group. We further found that this effect was driven by posterior CA2/3. By contrast, the semantic details from autobiographical memories, and performance on a range of laboratory-based memory tests, did not correlate with CA2/3 volume. Similarly, no associations with subfield volume were identified for the higher performing group.

There are only two published studies that have examined the relationship between hippocampal subfield volumes and autobiographical memory recall^28,29^. Our findings broadly align with those of Palombo et al.^29^ who, studying only the body of the hippocampus, found that the volume of a combined ROI comprising the DG/CA4/CA2/3 was associated with internal details scores. We can now extend this previous result in four key ways. First, because we had such a large sample, we were able to ascertain that an association between subfield volume and autobiographical memory ability was only evident in lower performing individuals, and not in those whose performance was above the median. Palombo et al.’s^29^ n = 30 may not have been sufficient to expose this difference. Our CA2/3 effect could not be explained by age, IQ, gender ratios or scanners used, which did not differ between groups. Second, we were more spatially precise in pinpointing CA2/3 as the key subregion, rather than a less informative, larger ROI. Third, because we segmented the entire length of the hippocampus, we showed that the effects were actually driven by posterior CA2/3. This is important because subfields differ in their connectivity along the anterior–posterior axis of the hippocampus^11,19,20,33,35^. Fourth, by examining semantic details from the autobiographical memories and a range of other laboratory-based memory tests, we ascertained that the relationship with CA2/3 volume was specific to autobiographical memory recall.

We also note that the sub-category of internal details that was most associated with bilateral posterior CA2/3 volume was “events”. It just failed to reach statistical significance when corrected for multiple comparisons. The event category contains details regarding happenings or the unfolding of the story, including individuals present, actions and reactions. As these are the core details at the heart of autobiographical memories, it is perhaps no surprise that this category seems to be the most associated with bilateral posterior CA3 volume in lower performing participants.

Our findings may suggest that lower performing individuals benefit most from greater posterior CA2/3 volume, with higher performing people not displaying increased volume. This could reflect the brain’s need to balance resources, because increasing the volume of a brain area can come at a cost. For example, in licensed London taxi drivers, their highly enhanced navigational skills and larger posterior hippocampal volume (compared to controls) was found to be accompanied by less volume in the anterior hippocampus^52,53^ and poorer performance on some anterograde visuospatial memory tests^54^. In the general population, it could be that when an individual’s autobiographical memory recall ability is at a certain level, there is sufficient functionality such that further increases in CA2/3 volume are no longer beneficial because of the potential costs involved. Our findings also suggest that it might be informative to specifically examine the volume of posterior CA2/3 in people who have been characterised as having “severely deficient autobiographical memory”^2^. These are individuals who are otherwise healthy but have poor autobiographical memory recall, primarily in terms of reduced visualisation of their autobiographical memories, and lower numbers of internal details when recalling memories from their childhood or teenage years. The prediction might be that their CA2/3 volume would be the most reduced among healthy controls.

In terms of patients, recent and remote autobiographical memories have been found to be susceptible to a loss of internal details in the context of focal bilateral damage to CA3^23^. Moreover, graph theoretic analyses of 7 T resting-state fMRI data revealed that the CA3 damage in these patients disrupted functional integration across the medial temporal lobe (MTL) subsystem of the default network. The loss of functional integration in MTL subsystem regions was predictive of autobiographical memory retrieval performance in the patients. Overall, these findings suggest that CA3 is necessary for the retrieval of autobiographical memories long after their initial acquisition, and that it plays a role in functional integration across the network of brain areas that is typically implicated in autobiographical episodic recall.

In the patients, CA3 damage was along the length of the hippocampus. The one fMRI study that examined representations of autobiographical memories in hippocampal subfields was able to nuance this further^25^. Remote autobiographical memories were particularly represented in posterior CA3. Our finding of specifically posterior CA2/3 volume correlating with autobiographical memory aligns with this previous result, at least in lower performing individuals. Of note, likely all of the memories elicited during the Autobiographical Interview^40^ are remote autobiographical memories. Even the most recent time period is “within the last year” with such memories also likely to have already undergone consolidation. The results were broadly similar when each time period was examined separately with respect to posterior CA2/3 volume, and no single time period reached statistical significance when corrected for multiple comparisons. Given that there is mostly only one memory for each time period, these analyses are likely underpowered compared to our main analyses, where the memories are grouped together.

What might CA2/3 be doing in the service of autobiographical memory recall, and why is its posterior portion particularly implicated? We cannot address these questions directly with our data, but we can offer some speculations. CA3 is thought to be a vehicle for pattern completion^30–32,55,56^, which involves the reinstatement of a memory trace from a partial cue. This computation clearly has relevance for autobiographical memory recall. No neuroimaging study has explicitly examined how pattern completion relates to autobiographical memories. However, larger CA2/3 volume has been associated with less neural overlap within CA3 between representations of similar memories of short movie clips, which in turn was associated with less subjective confusion during recall^57^. The authors of that study suggested that a larger CA3 may promote a decrease in retrieval confusion via an increased number of CA3 neurons, or enhanced lateral connectivity within CA3, either of which could precipitate more efficient pattern completion. It may be that a similar situation is playing out in our lower performing group, with greater CA2/3 volume facilitating better pattern completion.

There is still debate about how functions differ down the long axis of the human hippocampus. It has been suggested that the anterior portion of the hippocampus may be involved in constructing visual scenes that form the basis of autobiographical memory recall, with posterior hippocampal regions populating the scene with specific details^26,58^. Indeed, the involvement of specifically posterior CA2/3 in remote autobiographical memory recall^25^ may reflect the increased pattern completion required to populate remote autobiographical memories. Another perspective argues that the posterior hippocampus supports fine-grained local representations, compared to large-scale global representations in the anterior hippocampus^26,34,58^. Consequently, our posterior CA2/3 result may reflect the increased capacity to support more detail during autobiographical memory recall. It would be interesting in future studies to examine autobiographical memory ability with respect to such local and global processing, and also in terms of functional connectivity^18,19,59^, which may provide further insights about the information flow in and out of posterior CA2/3 during autobiographical memory recall.

Our CA2/3 findings only related to autobiographical memory recall, with performance on most of the other memory tests not differing between lower and higher performing groups. The one test on which the groups diverged was the immediate and delayed recall of the Logical Memory (story recall) test, perhaps the closest laboratory-based memory test to autobiographical memory retrieval. The lower performing group had poorer scores on this test compared to the higher performing group. However, there were no relationships in the lower performing group between posterior CA2/3 volume and either immediate or delayed recall on this test. This may reflect the particular association of the posterior hippocampus with the recall of remote memories (experienced many months or years before) rather than the very recently experienced story in the Logical Memory test^25^. In addition, the posterior CA2/3 volume in lower performers may be associated with the vivid, detailed and multimodal nature of autobiographical memory recall, as opposed to the simpler nature of the more constrained laboratory-based memory tests. This possible explanation aligns with the findings of a fMRI meta-analysis^41^, and a recent investigation into white matter microstructure^42^. Both of these studies showed that the recall of autobiographical memories and laboratory-based memory stimuli were associated with distinct neural substrates. More generally, our findings add further support to the increasingly-recognised importance of studying real-world cognition in order to fully characterise brain-behaviour relationships^60–66^.

There are a number of caveats that need to be borne in mind when interpreting our findings. First, our results suggest that there is not a simple linear relationship between posterior CA2/3 volume and autobiographical memory ability. For example, while posterior CA2/3 volume correlated with autobiographical memory recall in the lower performing group, it was not the case that CA2/3 volume increased in that group and then plateaued in the higher performing group. Indeed, no differences were identified in mean CA2/3 volumes in the lower and higher performing groups, nor where there any differences in mean CA2/3 volumes between the extremes of the highest and lowest performers. Furthermore, after dividing the sample into two groups based on the median posterior CA2/3 volume, we found no relationship between volume and autobiographical memory recall ability in either group. This means that an individual with exceptional autobiographical memory recall ability could have a similar posterior CA2/3 volume as someone with much poorer recall. In short, knowing a person’s posterior CA2/3 volume cannot necessarily inform about their autobiographical memory ability. Clearly, further research is needed to elucidate this complex pattern of findings further.

Second, we also predicted that the volume of the subiculum^29^ and/or anterior pre/parasubiculum^26,28,33^ might be associated with autobiographical memory recall ability. However, this hypothesis was not supported by the data. This does not mean that the subiculum, pre/parasubiculum, or indeed any of the other subfields, are not necessary for autobiographical memory recall, merely that their volume may not be indicative of underlying factors driving individual differences. A study that examined the relationship between hippocampal subfield volumes and autobiographical memory recall ability found that pre/parasubiculum volume was associated with better autobiographical memory persistence over an eight month delay^28^. This longitudinal study allowed for a focus on memory retention over time, which was not possible to investigate in the current experiment, precluding a direct comparison.

Third, in the current study, we tested a large sample of 201 participants. However, even with this sample size we cannot rule out the existence of particularly small effects that might indicate a significant association, such as that seen between CA2/3 and autobiographical memory recall when examining our sample as a whole (r(193) = 0.10, p = 0.15). Indeed, for this relationship to reach statistical significance, a sample of at least 500 participants would be needed, currently requiring 300 h of MRI scan time and 4000 h of manual hippocampal subfield segmentation. Future work seeking to relate individual differences in autobiographical memory to brain structure and function might consider adopting the consortium and “mega-analysis” approaches of, for example, the genetics literature^67^. In addition, there is an urgent need for accurate automated subfield segmentation methods that segment the hippocampus with precision along its entire length, including separating DG and CA2/3, and subiculum and pre/parasubiculum.

Fourth, while the median split approach is frequently used in the published literature, it is not without limitations. Performing a median split results in the formation of two smaller groups and a consequent reduction in power. Furthermore, median splits separate individuals who are just above and just below the median, even though these participants are likely quite similar. Ideally, allocation to lower and higher performing groups here would have been performed using an independent measure of autobiographical memory recall ability, however, there is a dearth of good quality measures.

Fifth, it should also be noted that the inter-rater agreement for delineating CA2/3 was lower than for most of the other subfields, highlighting the challenge of studying this small area. However, the values are similar to those reported in previous studies^19,25,28,57,68^, and are within the range defined as showing “good” reliability in the literature, with larger subfields having “excellent” reliability^69^. We are confident, therefore, in our CA2/3 delineations, but this point should be borne in mind when interpreting the results.

Sixth, well-powered individual differences studies relating autobiographical memory to brain structure and function remain rare. The analysis of other aspects of data from the cohort reported here identified no relationships between whole, anterior or posterior hippocampal grey matter volume or grey matter microstructure (such as myelination and iron) with autobiographical memory recall ability^8,43^. On the other hand, specific white matter microstructure features related to axonal conduction velocity were associated with individual differences in autobiographical memory recall ability, but not laboratory-based memory stimuli^42^. Investigations using other large samples have identified relationships between temporal pole volume and autobiographical memory recall in healthy older adults^70^ and different patterns of resting state functional connectivity in both younger and older adults between autobiographical memory recall and a control measure^59^. Investigations such as these, along with further studies of hippocampal subfields, will hopefully help to build a much more complete understanding of why such large variability exists within the general population in their capacity to recall their everyday experiences. We also note that there are different approaches to defining the optimal metric of autobiographical memory recall ability^59,70^ and control measures^29,44,45^ from the Autobiographical Interview^40^. Future work could compare these different methods and how they relate to hippocampal subfield volumes.

Finally, the current study focused specifically on young, healthy individuals. It is possible that variations in subfield volumes due to aging or disease might have different relationships with autobiographical memory performance. Future hippocampal subfields studies should examine individual differences in these types of cohorts in addition to young healthy individuals.

In conclusion, we examined relationships between the volumes of manually segmented hippocampal subfields of healthy people and their autobiographical memory recall ability in the largest such sample reported to date. While our findings highlight that posterior CA2/3 may be particularly pertinent for autobiographical memory recall, they also reveal that there may not be direct one-to-one mapping of posterior CA2/3 volume with autobiographical memory ability, with size mattering perhaps only in those with poorer memory recall.

## Methods

### Participants

Two hundred and one healthy people were included in the study, 104 females and 97 males. As detailed in previous publications^8,42,43^, the age range was restricted to 20–41 years old to limit the possible effects of aging (mean age = 29.05 years, *SD* = 5.65). Participants had English as their first language and reported no history of psychological, psychiatric or neurological conditions. People with hobbies or vocations known to be associated with the hippocampus (e.g. licenced London taxi drivers) were excluded. Two hundred and seventeen participants were initially recruited, however, the scan quality for sixteen participants was too poor for accurate hippocampal segmentation and they were subsequently excluded. Participants were reimbursed £10 per hour for taking part which was paid at study completion.

All participants gave written informed consent, and the study was conducted in accordance with the approval of the University College London Research Ethics Committee (project ID: 6743/001). All methods were performed in accordance with the relevant guidelines and regulations.

A sample size of over 200 participants was determined during study design to be appropriate as it is robust to employing different statistical approaches when answering multiple questions of interest. Specifically, the sample allowed for sufficient power to identify medium effect sizes when conducting correlation analyses at alpha levels of 0.01 and when comparing correlations at alpha levels of 0.05^71^. Samples of over 200 participants have also been suggested as appropriate for correlational neuroimaging research similar to that performed here^36,37^.

### The autobiographical interview

This widely-used test^40^ was employed to measure autobiographical memory recall ability. Participants are asked to provide autobiographical memories from a specific time and place over four time periods—early childhood (up to age 11), teenage years (aged from 11 to 17), adulthood (from age 18 years to 12 months prior to the interview; two memories are requested) and the last year (a memory from the last 12 months); therefore, five memories in total are harvested. Recordings of the memory descriptions are transcribed for later scoring.

The main outcome measure of the Autobiographical Interview is the mean number of internal details included in the description of an event from across the five autobiographical memories. Internal details are those describing the event in question (i.e. episodic details) and include event, place, time and perceptual information, as well as thoughts and emotions relating to the event itself. We used the mean number of semantic details included in the five autobiographical memories, as a control measure. Semantic details pertain to semantic information about or related to the past event, and are not considered to reflect autobiographical memory recall ability.

Double scoring was performed on 20% of the data. Inter-class correlation coefficients, with a two-way random effects model looking for absolute agreement were calculated for both internal and semantic details. This was performed both for individual memories and as an average of all five memories across each participant. For internal details the coefficients were 0.94 and 0.97 respectively, and for semantic details they were 0.80 and 0.84 respectively. For reference, inter-class correlation coefficients between 0.75 and 0.90 are considered to have good reliability, and inter-class correlation coefficients of 0.90 and above are considered to have excellent reliability^69^.

### Laboratory-based tests

Estimates of participants' IQ was obtained from the Test of Premorbid Functioning^46^. The number of correct responses was converted to an estimate of Full Scale IQ (FSIQ) as per the standard scoring procedure.

Eight laboratory-based memory tests were also administered to participants. These were memory tests that are often used in neuropsychological settings. Tasks were performed and scored in line with their standardised and published protocols. Specifically:

Verbal list recall was assessed using the immediate and delayed recall of the Rey Auditory Verbal Learning Test^47^. Visuospatial recall was examined using the delayed recall of the Rey–Osterrieth Complex Figure^48^. Recognition memory was investigated using the Warrington Recognition Memory Tests for Words and Faces^49^. Participants also underwent the “Dead or Alive” task which probes general knowledge about whether famous individuals have died or are still alive, providing a measure of semantic memory^50^. Finally, the ability to recall a short narrative was examined using the immediate and delayed recall of the Logical Memory subtest of the Wechsler Memory Scale IV^51^.

Note that the autobiographical memory recall data and the data from the laboratory-based tests reported here were included in a previous principal component analysis which sought to examine the structure of these behavioural data—see Clark et al.^72^ for further details.

### MRI data acquisition

As detailed in previous publications^8,42,43^, three MRI scanners were used to collect the neuroimaging data. All scanners were Siemens Magnetom TIM Trio systems with 32 channel head coils and were located at the same neuroimaging centre, running the same software. The sequences were loaded identically onto the individual scanners. Participant set-up and positioning followed the same protocol for each scanner.

Participants were scanned using a structural MRI sequence which was optimized for high-resolution imaging of the hippocampus. Data were collected using a single-slab 3D T2-weighted turbo spin echo sequence with variable flip angles^73^ in combination with parallel imaging to simultaneously achieve a high image resolution of ~ 500 μm, high sampling efficiency, and short scan time while maintaining a sufficient signal-to-noise ratio. After excitation of a single axial slab, the image was read out with the following parameters: resolution = 0.52 × 0.52 × 0.5 mm, matrix = 384 × 328, partitions = 104, partition thickness = 0.5 mm, partition oversampling = 15.4%, field of view = 200 × 171 mm, echo time (TE) = 353 ms, TR = 3200 ms, GRAPPA × 2 in phase-encoding (PE) direction, bandwidth = 434 Hz/pixel, echo spacing = 4.98 ms, turbo factor in PE direction = 177, echo train duration = 881, averages = 1.9. For reduction of signal bias due to, for example, spatial variation in coil sensitivity profiles, the images were normalised using a pre-scan, and a weak intensity filter was applied as implemented by the scanner's manufacturer. To improve the signal-to-noise ratio of the anatomical image used for segmentation, three scans were acquired for each participant, with a total scanning time of 39 min. Each structural scan was visually inspected for quality. Where scan quality was compromised due to movement artefacts, it was discarded. High quality scans for each participant were co-registered, denoised and averaged.

### Segmentation of hippocampal subfields

For each participant, we manually delineated hippocampal subfields, bilaterally, on the native space averaged and denoised high resolution structural image according to the methodology described by Dalton et al.^22^ using the ITK Snap software version 3.2.0. Masks were created for the following subregions: DG/CA4, CA2/3, CA1, subiculum, pre/parasubiculum and uncus (Fig. 1). Subfield segmentations were conducted by two segmenters (I.A.C. and M.A.D).

Reliability of the hippocampal segmentations was assessed using inter- and intra-rater reliability measures. Our main focus was on inter-rater reliability, with each researcher independently segmenting both hippocampi of the same 20 participants (10% of the total). As hippocampal segmentation took approximately 8 h per participant, segmentation of the full sample was performed over a period of 3.5 years (from July 2018 to December 2021). Independent hippocampal segmentations to assess reliability were performed throughout this time period, serving also to provide a measure of consistency over time; were either researcher to deviate from the segmentation protocol over the 3.5 years, then we would know this because inter-rater measures would reduce.

Reliability analyses were performed for each subfield using the Dice overlap metric^74^ which produces a score between 0 (no overlap) and 1 (perfect overlap). Inter-class correlation coefficients were also computed (using a two way random effects model looking for absolute agreement), where inter-class correlation coefficients between 0.75 and 0.90 are considered to have good reliability and inter-class correlation coefficients of 0.90 or above are considered to have excellent reliability^69^.

Inter-rater reliability metrics were equivalent to those reported in the extant literature^19,25,28,57,68,75–77^. Dice inter-rater reliability was 0.85 for DG/CA4, 0.68 for CA2/3, 0.78 for CA1, 0.80 for subiculum, 0.70 for pre/parasubiculum and 0.83 for the uncus. Inter-class correlation coefficients were 0.91 for DG/CA4, 0.75 for CA2/3, 0.91 for CA1, 0.90 for subiculum, 0.76 for pre/parasubiculum and 0.97 for the uncus. The results for each of the 20 individual Dice inter-rater reliability analyses (including the date of each segmentation) are provided in Supplementary Table S7. At all time points, Dice inter-rater reliability scores were in line with previous time points and publications, demonstrating high levels of consistency over the 3.5 year period, as well as high reliability between the two segmenters.

Intra-rater reliability was also high for both segmenters. For I.A.C., Dice intra-rater reliability (over 5 participants, measured on average 32.8 months (SD = 13.22) apart was 0.91 for DG/CA4, 0.81 for CA2/3, 0.86 for CA1, 0.86 for subiculum, 0.80 for pre/parasubiculum and 0.89 for the uncus, with inter-class correlation coefficients of 0.96 for DG/CA4, 0.93 for CA2/3, 0.85 for CA1, 0.99 for subiculum, 0.83 for pre/parasubiculum and 0.97 for the uncus. For M.A.D. (as previously reported in Ref.^22^.), Dice intra-rater reliability (over 6 participants, measured 3 months apart) was 0.86 for DG/CA4, 0.76 for CA3/2, 0.85 for CA1, 0.86 for subiculum, 0.75 for pre/parasubiculum and 0.87 for the uncus with inter-class correlation coefficients of 0.91 for DG/CA4, 0.84 for CA2/3, 0.89 for CA1, 0.95 for subiculum, 0.72 for pre/parasubiculum and 0.96 for the uncus.

Each subfield, with the exception of the uncus, was also divided into its anterior and posterior portions^8,34^. In line with literature^38,39^, the anterior was defined as proceeding from the first slice where the hippocampus can be observed in its most anterior extent until the final slice of the uncus (the uncal apex), and the posterior hippocampus was defined from the first slice following the uncal apex until the final slice of observation in its most posterior extent (Fig. 1).

### Statistical analyses

Analyses were performed in R 4.1. Data were summarised using means and standard deviations.

In our main analyses, we investigated the relationships between each subfield volume and the number of internal details from the Autobiographical Interview using partial correlations, with bootstrapping performed 10,000 times to calculate confidence intervals. The packages ppcor v1.1^78^ and RVAideMemoire v0.9.81.2 were utilised to do this. Six covariates were included in each partial correlation: age, gender, full scale IQ, scanner, total hippocampal volume and total intracranial volume. For the analyses investigating male and female participants separately, gender was not included as a covariate. For analyses investigating anterior and posterior subfield volumes, total anterior or posterior hippocampal volume was included as a covariate instead of total hippocampal volume. Control analyses were performed identically but subfield volume was correlated with either the total number of semantic details from the Autobiographical Interview or the Logical Memory immediate or delayed recall scaled scores.

Where comparison was made between internal details and control correlations, statistical differences were tested for using the technique described by Meng et al.^79^. This approach extends the Fisher z transformation, allowing for more accurate testing and comparison of two related correlations. The correlation comparison was performed using the R cocor package v1.1.3^80^. As the comparison of correlations was performed only when a significant correlation has been previously identified (Bonferroni corrected at p < 0.0042—see main text), a two-sided *p*-value < 0.05 was deemed significant.

Participants were also allocated to lower or higher performance groups depending on their scores compared to the median number of internal details of the whole sample. If their total number of internal details was less than or equal to the median they were assigned to the lower performing group (n = 101), whereas scores greater the median resulted in allocation to the higher performing group (n = 100). In addition, the 20 participants with the highest number of internal details were allocated to the very highest performing group, and the 20 lowest scoring were allocated to the very lowest performing group. Comparisons of the hippocampal subfield volumes between the lower and higher performing groups, and between the very highest and very lowest performers were made using one way univariate ANCOVAs with the same covariates as the partial correlations, using the R package car v3.0-12^81^.

The comparisons of the lower and higher performing groups on the other measures (Table 4) were performed using t-tests for continuous variables or chi-square tests for categorical variables. Differences were deemed significant following a two-sided *p*-value < 0.05.

## Supplementary Information

Supplementary Tables.

Supplementary Information 2.

## Data Availability

The data analysed during this study are included in the Supplementary Dataset file.
