# Supplementary material for: Posterior hippocampal CA2/3 volume is associated with autobiographical memory recall ability in lower performing individuals
Source: Sci Rep. 2023 May 16;13:7924. doi: 10.1038/s41598-023-35127-2 (PMC10188443; doi:10.1038/s41598-023-35127-2)
Supplement: Supplementary file 1 — Supplementary Tables. [file 41598_2023_35127_MOESM1_ESM.pdf]

Supplementary Information

Posterior hippocampal CA2/3 volume is associated with autobiographical memory recall ability in lower performing individuals

Ian A. Clark, Marshall A. Dalton, Eleanor A. Maguire

Table S1. Mean volumes and partial correlations - autobiographical memory recall ability (internal details) and bilateral <u>anterior</u> hippocampal subfield volumes across the whole group.

Hippocampal subfield	Mean (SD) volume (mm^3)	r(193)	p	95% Confidence interval	
				Lower	Upper
Anterior DG/CA4	347.18 (89.88)	-0.02	0.80	-0.16	0.12
Anterior CA2/3	97.20 (27.11)	0.05	0.51	-0.09	0.19
Anterior CA1	420.94 (89.62)	-0.04	0.59	-0.19	0.09
Anterior subiculum	454.20 (87.61)	0.10	0.17	-0.04	0.24
Anterior pre/parasubiculum	227.76 (48.03)	0.11	0.13	-0.03	0.24
Uncus	888.54 (192.09)	-0.03	0.66	-0.17	0.11

Threshold for statistical significance is $p < 0.0042$ (Bonferroni correction for multiple comparisons).

Table S2. Mean volumes and partial correlations - autobiographical memory recall ability (internal details) and bilateral <u>posterior</u> hippocampal subfield volumes across the whole group.

Hippocampal subfield	Mean (SD) volume (mm^3)	r(193)	p	95% Confidence interval	
				Lower	Upper
Posterior DG/CA4	795.54 (127.65)	-0.09	0.22	-0.23	0.05
Posterior CA2/3	204.06 (37.10)	0.13	0.07	-0.01	0.26
Posterior CA1	818.48 (120.24)	-0.09	0.19	-0.23	0.05
Posterior subiculum	655.96 (117.30)	0.07	0.34	-0.07	0.21
Posterior pre/parasubiculum	313.83 (53.66)	0.08	0.27	-0.06	0.22

Note. There is no uncus in the posterior hippocampus. Threshold for statistical significance is $p < 0.0042$ (Bonferroni correction for multiple comparisons).

Table S3. Partial correlations between <u>bilateral</u> CA2/3 volume and each of the <u>internal detail sub-categories</u> in the lower performing participants.

Internal detail sub-category	r(93)	p	95% Confidence interval	
			Lower	Upper
Event	0.18	0.08	-0.02	0.37
Place	0.11	0.27	-0.09	0.31
Time	0.13	0.20	-0.07	0.32
Perceptual	0.16	0.11	-0.04	0.35
Emotion/thought	0.25	0.01	0.05	0.43

Threshold for statistical significance is $p < 0.0042$ (Bonferroni correction for multiple comparisons).

Table S4. Partial correlations in the lower performing participants between bilateral CA2/3 volume and autobiographical memory recall ability at each of the four time periods included in the Autobiographical Interview.

Time Period	r(93)	p	95% Confidence interval	
			Lower	Upper
Early childhood	0.10	0.33	-0.10	0.30
Teenage years	0.25	0.01	0.05	0.43
Adulthood	0.22	0.03	0.02	0.40
Last year	0.25	0.01	0.05	0.43

Threshold for statistical significance is $p < 0.0042$ (Bonferroni correction for multiple comparisons).

Table S5. Partial correlations between bilateral posterior CA2/3 volume and each of the internal details sub-categories in the lower performing participants.

Internal detail sub-category	r(93)	p	95% Confidence interval	
			Lower	Upper
Event	0.28	0.0059	0.08	0.46
Place	0.09	0.39	-0.11	0.29
Time	0.14	0.18	-0.06	0.33
Perceptual	0.15	0.16	-0.06	0.34
Emotion/thought	0.25	0.01	0.06	0.43

Threshold for statistical significance is $p < 0.0042$ (Bonferroni correction for multiple comparisons).

Table S6. Partial correlations in the lower performing participants between posterior CA2/3 volume and autobiographical memory recall ability at each of the four time periods included in the Autobiographical Interview.

Time Period	r(93)	p	95% Confidence interval	
			Lower	Upper
Early childhood	0.11	0.27	-0.09	0.31
Teenage years	0.22	0.04	0.01	0.40
Adulthood	0.26	0.01	0.06	0.44
Last year	0.19	0.06	-0.01	0.38

Threshold for statistical significance is $p < 0.0042$ (Bonferroni correction for multiple comparisons).

Table S7. Individual Dice inter-rater reliability results for all 20 reliability segmentations, shown in date order of segmentation.

Subfield	IAC: Aug-18 MAD: July-18	IAC: Sept-18 MAD: July-18	IAC: Sept-18 MAD: July-18	IAC: Sept-19 MAD: May-20	IAC: Sept-19 MAD: May-20
DG/CA4	0.85	0.86	0.87	0.84	0.83
CA2/3	0.70	0.70	0.73	0.70	0.63
CA1	0.79	0.79	0.77	0.80	0.78
Subiculum	0.80	0.79	0.79	0.82	0.79
Pre/para	0.75	0.72	0.69	0.75	0.68
Uncus	0.85	0.82	0.81	0.82	0.80

	IAC: March-20 MAD: May-20	IAC: April-20 MAD: May-20	IAC: April-20 MAD: May-20	IAC: May-20 MAD: June-20	IAC: June-20 MAD: June-20
DG/CA4	0.83	0.83	0.84	0.85	0.84
CA2/3	0.69	0.65	0.68	0.63	0.69
CA1	0.80	0.78	0.79	0.79	0.79
Subiculum	0.80	0.80	0.81	0.78	0.81
Pre/para	0.70	0.70	0.75	0.67	0.70
Uncus	0.81	0.84	0.86	0.82	0.85

	IAC: July-20 MAD: Sept-20	IAC: Nov-20 MAD: Nov-20	IAC: Nov-20 MAD: Nov-20	IAC: Nov-20 MAD: Nov-20	IAC: Feb-21 MAD: Feb-21
DG/CA4	0.88	0.86	0.87	0.86	0.87
CA2/3	0.71	0.67	0.60	0.69	0.66
CA1	0.82	0.80	0.73	0.77	0.82
Subiculum	0.82	0.80	0.77	0.83	0.82
Pre/para	0.68	0.72	0.70	0.70	0.71
Uncus	0.82	0.84	0.84	0.82	0.85

	IAC: March-21 MAD: April-21	IAC: May-21 MAD: June-21	IAC: July-21 MAD: July-21	IAC: Oct-21 MAD: July-18	IAC: Oct-21 MAD: July-18
DG/CA4	0.83	0.86	0.86	0.83	0.86
CA2/3	0.66	0.67	0.70	0.70	0.65
CA1	0.78	0.76	0.78	0.76	0.79
Subiculum	0.80	0.80	0.82	0.78	0.79
Pre/para	0.68	0.67	0.70	0.68	0.70
Uncus	0.84	0.84	0.85	0.82	0.82

IAC = segmenter Ian A. Clark; MAD = segmenter Marshall A Dalton; Pre/para = Pre/parasubiculum.
